# Pre-hospital data as risk predictors of seriousness among traumatically injured patients

**DOI:** 10.1186/1757-7241-23-S1-A3

**Published:** 2015-07-16

**Authors:** Alexander Franzén, Kim L Schultz, Jens O Laursen, Christian B Mogensen

**Affiliations:** 1 Emergency Department, Hospital of Southern Jutland, Aabenraa, Denmark

## Background

Currently many hospitals activate a trauma team with a predefined large team of health care professionals as a response to a trauma call. For hospitals with limited resources, this is a demanding process, which weakens the overall hospital performance by allocating considerable resources to the trauma room. Most trauma calls are based on trauma schemes scores from a combination of physiologic- anatomic injury- and injury mechanism criteria. In the search for indicators which might be used for a more differentiated hospital response, the aim of this study was to investigate the relative importance of pre-hospital variables in identifying "high risk" patients.

## Methods

The study was a historical prospective cohort study conducted at a level 2 trauma hospital in Southern Denmark. The inclusion criterion was traumatically injured patients above 14 years of age, requiring activation of the trauma team over a one-year period. The outcome was "high risk" patients, requiring one or more of the following: In-hospital stay more than 48 hours, orthopaedic or non-orthopaedic surgery performed, ICU stay, transfer to another hospital, or injury related death within 30 days. Logistic regression was used to evaluate the relationship between pre-hospital variables and high risk.

## Results

Of the 393 injured patients included, 30.0% were high risk patients. Statistically significant independent variables associated with high risk included anatomic injury criteria (OR = 5.5; 95% CI: 2.13-14.23), age 35-55 years (OR = 2.7; 95% CI: 1.31-5.55), age above 55 years (OR = 4.8; 95% CI: 2.30-9.97), pre-hospital systolic blood pressure 90-110 mmHg (OR = 3.8; 95% CI 1.02-13.92), "pedestrian struck by motor vehicle" (OR = 4.3; 95% CI: 1.42-12.76), and oxygen saturation 90-94% (OR = 3.4; 95% CI: 1.30-8.64).

## Conclusions

Our findings demonstrate that age, systolic blood pressure, oxygen saturation, and anatomic injury criteria are associated with high risk traumas and should be considered for inclusion in a trauma team activation protocol and further tested in such a model. Besides pedestrians struck by motor vehicle, the mechanism of injury has revealed poor predictive capabilities.

